# The Role of VP16 in the Life Cycle of Alphaherpesviruses

**DOI:** 10.3389/fmicb.2020.01910

**Published:** 2020-08-18

**Authors:** Dengjian Fan, Mingshu Wang, Anchun Cheng, Renyong Jia, Qiao Yang, Ying Wu, Dekang Zhu, Xinxin Zhao, Shun Chen, Mafeng Liu, Shaqiu Zhang, Xumin Ou, Sai Mao, Qun Gao, Di Sun, Xingjian Wen, Yunya Liu, Yanling Yu, Ling Zhang, Bin Tian, Leichang Pan, Xiaoyue Chen

**Affiliations:** ^1^Institute of Preventive Veterinary Medicine, Sichuan Agricultural University, Chengdu, China; ^2^Key Laboratory of Animal Disease and Human Health of Sichuan Province, Sichuan Agricultural University, Chengdu, China; ^3^Avian Disease Research Center, College of Veterinary Medicine, Sichuan Agricultural University, Chengdu, China

**Keywords:** alphaherpesvirus, VP16, transcriptional activation, secondary envelopment, reactivation from latency

## Abstract

The protein encoded by the UL48 gene of alphaherpesviruses is named VP16 or alpha-gene-transactivating factor (α-TIF). In the early stage of viral replication, VP16 is an important transactivator that can activate the transcription of viral immediate-early genes, and in the late stage of viral replication, VP16, as a tegument, is involved in viral assembly. This review will explain the mechanism of VP16 acting as α-TIF to activate the transcription of viral immediate-early genes, its role in the transition from viral latency to reactivation, and its effects on viral assembly and maturation. In addition, this review also provides new insights for further research on the life cycle of alphaherpesviruses and the role of VP16 in the viral life cycle.

## Introduction

The *Herpesviridae* family is classified into three subfamilies, *alphaherpesvirinae*, *betaherpesvirinae*, and *gammaherpesvirinae*. For all herpesviruses, a complete virion consists of four parts: a core that contains a double-stranded DNA genome, a capsid, a tegument, and an envelope (Guiping et al., 2007; Boštíková et al., 2014). The *alphaherpesvirinae* subfamily includes herpes simplex virus type-1/2 (HSV-1/2), pseudorabies virus (PRV), duck enteritis virus (DEV) (Qi et al., 2008; Zhao et al., 2008; Chang et al., 2009; Guo et al., 2009; Jia et al., 2009), varicella-zoster virus (VZV), equine herpesviruses (EHV), bovine herpesvirus (BHV), canine herpesvirus (CHV), and Marek’s disease virus (MDV) (Kobty, 2015; Smith, 2017; You et al., 2017; He et al., 2018; Lefkowitz et al., 2018).

The UL48 gene is conserved in most alphaherpesviruses, encoding the late tegument protein VP16, also known as alpha-gene-transactivating factor (α-TIF), but is not conserved in *betaherpesvirinae* and *gammaherpesvirinae* (Zhang et al., 2016). In the early stage of viral infection, VP16 released by invading virions binds to the immediate-early (IE) gene promoter to stimulate the transcription of IE genes as a transactivating factor that acts specifically on IE genes. In the late stage, VP16 assembles into the tegument to participate in the assembly of virions and promote their maturation (Jonker et al., 2005b; Zhang et al., 2016). In recent years, research on VP16 has found that its function is powerful and involves complex regulatory networks (Zhang et al., 2016; Sevin-Pujol et al., 2017). This article will explain the role of VP16 in promoting IE gene transcription, as well as its role in viral assembly and how VP16 functions when the virus reactivates from latency, providing new insights into the maturation of alphaherpesviruses and the role of VP16 in the viral life cycle.

## The Life Cycle of Alphaherpesviruses

During herpesvirus infection, the virions adsorb to the plasma membrane through interactions between envelope glycoproteins and host cell-specific receptors, and the virions become engulfed in phagocytic vesicles, which derived from invaginating plasmalemma and enter host cells (Mettenleiter et al., 2006; Zhang et al., 2011). After entering the cell, the capsid and tegument gradually loosen and disintegrate, and the nucleocapsids are transported to the nuclear pore, releasing the viral DNA to the nucleus. Next, the DNA genome is circularized and replicated, and viral capsid proteins synthesized in the cytoplasm enter the nucleus to form capsids. Then, the viral genome is cleaved and packaged into the capsids to form nucleocapsids (Dai et al., 2013; Homa and Brown, 2015).

Nucleocapsids are translocated into the cytoplasm via a process of primary envelopment–deenvelopment: nucleocapsids bud into the inner nuclear membrane to obtain the primary envelope and enter the perinuclear space, then the primary envelope fuses with the outer nuclear membrane to deenvelop, and unenveloped capsids are released to the cytoplasm. Then, nucleocapsids can bind in an orderly manner to tegument proteins (mainly inner tegument proteins) and be transported through microtubules to *trans*-Golgi-derived vesicles that combine viral glycoproteins with outer tegument proteins, budding into vesicles via secondary envelopment to form complete virions. Eventually, the vesicles carry fully assembled virions to the plasma membrane for egress via the exocytosis pathway (Guo et al., 2010; Crump, 2018; Yang et al., 2019).

VP16 plays a role in mainly two phases of the viral life cycle. First, VP16 is a transcriptional activator that regulates viral gene transcription. Second, VP16 is a late tegument protein that further participates in the assembly and maturation of nucleocapsids in the cytoplasm.

## VP16 Is a Transcriptional Activator of IE Genes

**FIGURE 1:**
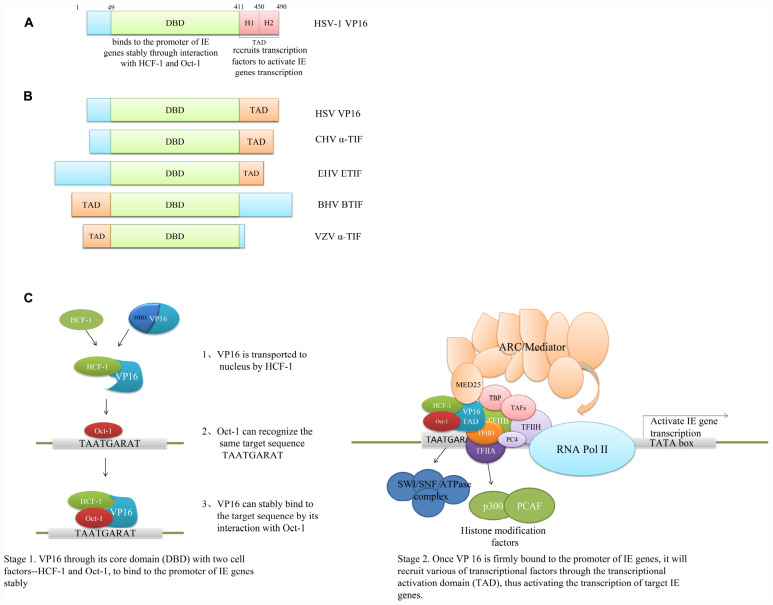
Domain structure and mechanism of VP16 to promote IE gene transcription. **(A)** Domain structure of HSV-1 VP16. The DBD can bind to the promoter of IE genes stably through interaction with HCF-1 and Oct-1, and the TAD of HSV VP16 can be divided into two regions, H1 and H2, which can bind to different transcriptional activators and also synergistically interact with some important transcriptional factors (Ikeda et al., 2002; Hirai et al., 2010). **(B)** The schematic of various VP16 homologs with their functional domains. The DBD is conserved in VP16 homologs, but the TADs of VP16 homologs are not conserved in position, sequence, or amino acid properties. The VP16 TAD of HSV and EHV is located at the C-terminus, but in BHV and VZV, the VP16 TAD is located at the N-terminus (Cohen and Seidel, 1994; Wysocka and Herr, 2003; Tyack et al., 2006). **(C)** Mechanism of VP16 to promote IE gene transcription. The DNA-binding ability of VP16 itself is weak, and the binding is unstable; VP16 mainly combines with HCF-1 and Oct-1 to form a complex through the unstructured region in its DBD, so as to bind stably to the specific site of the target gene promoter and then recruit transcriptional factors through the TAD to promote the transcription of the target gene. The formation of the complex by VP16, HCF-1, and Oct-1 is the most effective combination by VP16 dominated to activate viral gene cascade expression. The VP16-induced complex represents a regulatory switch for two modes of viral infection: lytic infection and latent infection. When it is “on,” it promotes the transcription of IE genes and thus lytic infection, and when it is “off,” limiting IE gene transcription, viruses can maintain established latent infections (Wysocka and Herr, 2003; Hirai et al., 2010; Milbradt et al., 2011).

After alphaherpesvirus infects target cells, the viral genome that enters the nucleus is transcribed in a specific order, IE genes, then early (E) genes, and finally late (L) genes (Nishiyama, 2006; Liu et al., 2015), and this cascade of transcription is precisely initiated by VP16 (Dalrymple et al., 1985; Lu and Misra, 2000). Once the host cell is infected, VP16 is released by the virions and together with two cell factors, HCF-1 and Oct-1, to form a transcriptional regulatory complex through its conserved DNA-binding domain (DBD), also named the VP16-induced complex-forming domain (VIC), which binds to the promoter of IE genes stably. Then, through its unconserved transcriptional activation domain (TAD), VP16 can recruit numerous transcription factors to activate the transcription of IE genes (Laboissière et al., 1997; Simmen et al., 1997) (Figure 1).

### The Formation of the VIC-Induced Transcriptional Regulatory Complex

The HSV VP16 VIC is located at residues 49–412, and the VIC contains both structural and non-structural regions (Liu et al., 1999). The structural region resembles a “seat-like” structure. A two-stranded antiparallel coiled coil formed by two long α-helixes is connected by a short six-amino-acid loop forming the back of the seat, two V-shaped spirals form the surface, and the seat bottom contains all of the strands (β1–β6) that form two β sheets: a two-stranded parallel β sheet (strands β5 and β2) and a four-stranded mixed antiparallel β sheet (strands β4, β3, β1, and β6) (Liu et al., 1999; Jonker et al., 2005a; Tyack et al., 2006). The seat surface can recognize and bind to the specific motif TAATGARAT on the IE gene promoter. Although VP16 can directly interact with the target sequence through the structured region, VP16 itself has weak DNA-binding ability; instead, it binds stably to DNA through the non-structural region (Lai and Herr, 1997; Babb et al., 2001; Tyack et al., 2006). The non-structural region is located mainly at residues 350–394 and 403–412, showing an irregular conformation. The residues 361–365 contain a specific motif (D/E)HXY(S/A) that can bind to HCF-1, and binding to Oct-1 is at mainly residues 373–378, but the formation of the transcriptional complex is also sensitive to changes in other parts of the non-structural region (Lai and Herr, 1997; Lu et al., 1998; Vogel and Kristie, 2013). VP16 itself does not possess a nuclear localization signal; it is transported to the nucleus by HCF-1 and then binds to Oct-1, which can also recognize the same target sequence. After binding, the irregular conformation of the non-structural region transforms into a stable and orderly structure, making VP16 firmly bind to the promoter of IE genes (Herr, 1998; Carroll et al., 2007; Liu et al., 2017) (Figure 1).

Serine at position 375 (Ser375) in VP16, also in the Oct-1-binding domain, resides in a CK2 site (S/TxxE/D) and can be phosphorylated by CK2. Both the CK2 site and Ser/Thr at position 375 are highly conserved among VP16 homologs (O’Reilly et al., 1997; Ottosen et al., 2006). Phosphorylation of Ser375 promotes the binding of VP16 to Oct-1, and replacing Ser375 with Thr (also phosphorylatable by CK2) but not Ala still results in Oct-1 binding to preserve the transcriptional activation by VP16. Other phosphorylation sites found in VP16 are Ser18, Ser353, and Ser452, and all of these sites are consistent with the target sequence for phosphorylation by kinases in the JNK families, but these sites are far less effective than Ser375 in VP16 transcriptional activation (Ottosen et al., 2006; Sawtell et al., 2011). This reflects the importance of the formation of a complex by VP16 and Oct-1 for transcriptional activation.

#### The Formation of a VIC-Induced Transcriptional Regulatory Complex

VP16 effectively binds to HCF-1 and Oct-1 through the kelch domain of HCF-1 and the POU domain of Oct-1 (Herr and Cleary, 1995; Luciano and Wilson, 2002). The kelch domain of HCF-1 is composed of six kelch repeats, and four-stranded β sheets formed by these repeats assemble into a six-bladed propeller-like structure, which can bind VP16 and maintain the stability of the VIC-induced complex (Wilson et al., 1997; Luciano and Wilson, 2002). The POU domain of Oct-1 consists of two independent DNA-binding units, the POU-specific (POU_S_) domain and the POU-homeo (POU_H_) domain, which are connected by an unstructured linker (Herr and Cleary, 1995), and VP16 is connected only to the POU_H_ domain (Chasman et al., 1999; Phillips and Luisi, 2000). The unstructured linker makes the combination of the Oct-1 POU subdomain with the DNA sequence very flexible, thus ensuring diversity of the DNA sequence recognition by Oct-1, which is why even IE-gene promoters of other alphaherpesviruses are not exactly TAATGARAT sequence but rather similar sequences, such as the TAATGAGCT motif in the BHV IE-1 promoter and the repeated TAATTACAC motifs in the CHV ICP4 promoter; Oct-1 can still bind to VP16 to form a VIC-induced complex on these promoters (Herr and Cleary, 1995; Phillips and Luisi, 2000; Wysocka and Herr, 2003; Tyack et al., 2006).

#### Forming the VIC-Induced Complex Is the Specific Combination to Activate IE Gene Transcription

The formation of the VIC-induced complex by VP16, HCF-1, and Oct-1 is the most effective combination of VP16 to activate the viral gene expression. In addition to HCF-1, the HCF family includes HCF-2, which can also stabilize the complex induced by VP16, but only the HCF-1-containing complex can effectively activate transcription (Johnson et al., 1999; Luciano and Wilson, 2002). In addition to the kelch domain, HCF-1 carboxy-terminal acidic region also contains a TAD, which cooperates with the activation domain of VP16 to activate transcription (Lee and Herr, 2001; Gudkova et al., 2019). Similarly, in addition to Oct-1, there are other proteins containing a POU domain. For example, in Oct-2, the protein most similar to Oct-1, only one amino acid is changed compared to Oct-1. Although they can recognize the same DNA sequence, Oct-2 cannot bind to VP16 (Cleary et al., 1993; Bentrari et al., 2015). The unique combination of VP16 with HCF-1 and Oct-1 provides evidence to explain why HSV can establish latent infection in sensory neurons: (1) as a cell proliferating factor, HCF-1 will change its status in non-proliferating cells to adapt to or even promote the cessation of cell proliferation. Moreover, HCF-1 exists in the cytoplasm of sensory neurons and is thus perhaps unable to transport VP16 to the nucleus and to play a role in transcription activation (Reilly and Herr, 2002; Wysocka and Herr, 2003). (2) There are many POU-domain proteins similar to Oct-1 in the nervous system, such as Oct-2, brn-1, and brn-2, that can effectively bind to the TAATGARAT site though they cannot interact with VP16 and may bind more tightly than Oct-1, thus perhaps inhibiting VP16-induced complex formation on the promoter and preventing gene transcription (Andersen and Rosenfeld, 2001; Wysocka and Herr, 2003). As a result, the VIC-induced complex represents a regulatory switch for two modes of viral infection: lytic infection and latent infection. When it is “on,” it promotes the transcription of IE genes and thus lytic infection, and when it is “off,” limiting IE gene transcription, viruses can maintain established latent infections (Wysocka and Herr, 2003; Thompson et al., 2009; Zhang et al., 2016).

### VP16 Activates IE Gene Transcription via Its TAD

#### The VP16 TAD Recruits Different Types of Transcription Factors

Once VP 16 is firmly bound to the promoter of IE genes, it will recruit transcription factors through its TAD, thus activating the transcription of target genes (Hall and Struhl, 2002) (Figure 1).

The VP16 TAD can interact with many different types of transcription factors. (1) VP16 can directly bind to common transcription factors, such as TFIIB, TFIIH, TATA-binding protein (TBP), and TBP-related factors (TAFs) (Goodrich et al., 1993; Klemm et al., 1995; Hori et al., 2004; Langlois et al., 2008). The ternary complex formed by VP16, TFIIA, and TFIID is the initiation complex of RNA polymerase II, which promotes the recruitment of RNA polymerase II and the correct initiation location (Kobayashi et al., 1995; Hori et al., 2004). (2) VP16 can also interact with the activator-recruited cofactor (ARC)/mediator coactivator complex, promoting the binding of additional transcription factors and RNA polymerase II to the target gene promoter (Aguilar et al., 2014). There are more than 30 known subunits that make up the ARC/mediator coactivator complex (Harper and Taatjes, 2018). VP16 binds to the coactivator complex by mainly interacting with MED25 (also named ARC92 or ACID1) or MED17 (Aguilar et al., 2014; Lee et al., 2018). (3) VP16 can also recruit chromatin modification factors and promote chromatin remodeling, which is divided into mainly two types. The first are histone modification factors, such as the histone acetyltransferases p300/CBP and PCAF, histone methyltransferase SUVH39H1 (methylation of lysine 9 on histone H3), and histone demethylase JMJD2A (demethylation of lysines 9 and 36 on histone H3) (Wang et al., 2000; Kutluay et al., 2009). Through covalent modification of histones, such as acetylation, methylation, and ubiquitination, the deposition of histones on promoters is prevented, and the structure of chromatin becomes loose, which facilitates the binding of transcription regulators with *cis*-acting elements and the sliding of RNA polymerase on transcription templates to promote gene transcription; histone acetylation is a symbol of transcriptional activation (Cantin et al., 2003; Herrera and Triezenberg, 2004). The other type is ATP-dependent chromatin remodeling complexes, such as the SWI/SNF ATPase complex, which makes nucleosomes slide along DNA through ATP hydrolysis and disrupts the order of nucleosomes, participating in DNA double-strand fracture repair and nucleotide removal repair and promoting transcriptional extension (Cantin et al., 2003; Wu et al., 2017). In the absence of binding partners, TAD does not have a specific three-dimensional structure, and when bound to interacting transcriptional factors, the TAD will undergo conformational transformation from irregular crimping to an α-helix, providing a strong electrostatic driving force to enhance binding to transcriptional factors (Jonker et al., 2005a; Langlois et al., 2008). Although binding to some basic transcription factors, histone acetyltransferases, and the SWI/SNF complex is a common feature of transcriptional activators, the TAD of VP16 is highly efficient and widely used. After replacing the TAD of the host transcriptional activator with the TAD of VP16, the transcriptional activation ability of the activator was significantly improved, which provides a new method for improving the activation efficiency of inefficient transcriptional activators (Langlois et al., 2008; Sevin-Pujol et al., 2017; Mittal et al., 2018; Wang et al., 2019).

#### Structural Characteristics of Different TADs of VP16

In alphaherpesviruses, the TADs of VP16 homologs are not conserved in position, sequence, or amino acid properties, but the TADs of transcriptional activators have a general feature that is a short sequence pattern with no positive charge but with redundant negative charge residues and aromatic residues (Tyack et al., 2006; Ravarani et al., 2018). The TAD of HSV-1 VP16 is composed of 80 amino acids at the C-terminus, two hydrophobic amino acid clusters, and adjacent acidic residues (Regier et al., 1993), and it is a highly acidic sequence in which hydrophobic and aromatic residues are critical for transcriptional activation, such as phenylalanine at sites 442, 473, 475, and 479. The TAD of HSV-1 VP16 can be divided into two regions: H1 (amino acids at residues 411–456) and H2 (amino acids at residues 450–490), which can bind to different transcriptional activators and synergistically interact with some important transcriptional factors (Regier et al., 1993; Sullivan et al., 1998). For example, effective binding of the major transcriptional factor TFIIB requires the joint participation of H1 and H2 (Walker et al., 1993), and interaction with MED25 depends mainly on H1 (Aguilar et al., 2014), while the effective connection with TFIIA, TFIID, and TFIIH occurs through H2 (Johnson and Carey, 2003; Fukuda et al., 2004). In EHV-1, the TAD of ETIF (a homolog of VP16) is only a small segment of the C-terminus, especially the last 7 amino acids, which are necessary to activate the transcription of IE genes. The ETIF TAD is essentially the same as the core of H2 (H2. CCR) of the HSV VP16 TAD. However, the HSV VP16 TAD does not affect the binding of the VP16 core domain to HCF-1 and Oct-1 to form the transcriptional regulatory complex, while ETIF relies only on the conserved core domain and cannot bind to HCF-1 and Oct-1. The absence of the ETIF TAD could destroy the conformation of the core domain and inhibit the formation of the transcriptional regulatory complex (Grapes and O’Hare, 2000). Methionine residues at the C-terminus are important for transcriptional activation by ETIF, and they are also found at the C-terminus of HSV VP16 and CHV VP16, but not in BTIF (the homolog of VP16) (Grapes and O’Hare, 2000; Tyack et al., 2006).

In contrast, the TAD of BTIF is located at the N-terminus, and when the N-terminus of HSV VP16 is replaced with the N-terminus of BTIF, it results in a recombinant chimeric protein with higher transcriptional activation activity than any α-TIF (Misra et al., 1994, 1995; Grapes and O’Hare, 2000). Similar to BTIF, the α-TIF of VZV, a product encoded by the ORF10 gene, also lacks the acidic C-terminus of VP16, whose TAD is located at the N-terminus (Cohen and Seidel, 1994) (Figure 1). These results indicate that although the VP16 homologs have a relatively conserved core domain, the N-terminal and C-terminal domains are very different, and the location and structure of the TAD of VP16 homologs are different. Even though the TAD has a similar structure, such as the ETIF TAD being a small part of the HSV VP16 TAD, it plays a very different role. Core domain conservation may ensure the formation of a similar VP16-dominated transcriptional regulatory complex that binds stably to IE gene promoters. However, with such a large difference in the VP16 TADs, the impact on the transcriptional factors recruited by different VP16 proteins and the recruitment methods remains to be further researched.

### The Transcriptional Activation Function of VP16 Is Influenced by Other Cellular or Viral Proteins

Some cellular proteins and viral proteins can regulate the transcription of IE genes mediated by VP16. Heat-shock protein 90α (Hsp90α) is a cellular molecular chaperone that not only promotes the nuclear transport of HSV-1 capsid proteins and the correct localization of DNA polymerase but also regulates the activity of the promoter of HSV IE genes and stimulates HSV-1 infection (Zhong et al., 2014). Hsp90α is not a direct activator of IE genes but can bind to the core domain of VP16, maintain the stability of VP16, and ensure that VP16 is not degraded by the autolysosomal degradation pathway, thus participating in the transcriptional activation of IE genes mediated by VP16 (Zhong et al., 2014; Wang et al., 2018). The viral tegument proteins pUL14, VP11/12 (encoded by UL46), and VP13/14 (encoded by UL47) can enhance the efficiency of IE gene transcription mediated by VP16, which may play the same role in promoting nuclear input of VP16 (Lee et al., 2008; Yamauchi et al., 2008; Hernández Durán et al., 2019). Another tegument protein, the protein kinase US3, can regulate the release of the virus and promote the dissociation of VP11/12, VP13/14, and VP16 from tegument by phosphorylation, allowing entry into the nucleus as early as possible to initiate the transcription of viral genes (Kato and Kawaguchi, 2018; Hernández Durán et al., 2019). The HSV-1IE protein ICP22 can inhibit the transcription of IE genes, but VP16 can relieve the inhibition. Although there is no direct interaction between VP16 and ICP22, ICP22 can interact with various transcription factors that also bind to VP16 (Cun et al., 2009; Guo et al., 2012). Therefore, the transcriptional regulation of IE genes by VP16 and ICP22 may be achieved through some unknown action of both and related transcription factors. What other cellular proteins or viral proteins are involved in regulating the transcriptional activation of IE genes mediated by VP16, and in addition to forming transcriptional regulatory complexes with HCF-1 and Oct-1, whether VP16 promotes the transcription of IE genes through other mechanisms are directions for future research on VP16.

## The Role of VP16 in Viral Assembly

VP16 is not only an efficient transcriptional activator in the life cycle of alphaherpesvirus, promoting gene transcription, but also an important tegument protein that can regulate the assembly and maturation of viruses.

### Effects of VP16 on the Proliferation and Replication of Different Alphaherpesviruses

Among alphaherpesviruses, VP16 has different effects on the effective proliferation and replication of the virus. In this regard, many studies have been performed by constructing different VP16-deleted mutants. The HSV strain SJO2 carries a mutation at Ser375 to Ala in VP16, which disrupts its interaction with Oct-1, leading to a decrease in the transcription level of the IE genes and a 10-fold reduction in viral titers (Ottosen et al., 2006; Sawtell et al., 2011). Another strain in 1,814, which inserts four amino acids after residue 379 of VP16, eliminates the transcriptional activation of the IE genes. At a low multiplicity of infection, the plaque-forming efficiency of the virions is reduced by 100–10,000-fold, and at high multiplicity, although it is not easy to form plaques, many virions can be detected (Preston et al., 1997; Smiley and Duncan, 1997). The whole TAD truncation mutation in strain RP5 also completely inhibits the transcriptional activation of VP16, but the viral titer decreases even more (Herrera and Triezenberg, 2004). These studies showed that the elimination of transcriptional activation of IE genes mediated by VP16 does not block production of mature virions but can greatly reduce the infectivity of generated virions. In addition to mediating IE gene expression, the VP16 TAD also has a non-transcriptional activation function that affects the production of virions. Another HSV mutant, 8MA, which lacks almost the entire VP16 ORF, could not produce detectable progeny viruses in non-complementary cells without VP16 and could not replicate, while 8MA-R (a revertant of 8MA) showed completely restored replication ability, and 8MA could replicate effectively only in complementary cell lines containing VP16 (Weinheimer et al., 1992; Mossman et al., 2000) (Table 1), indicating that VP16 is necessary for HSV proliferation and replication. Similarly, DNA transfection of an ETIF deletion mutant (vL11ΔETIF) resulted in only transient expression and no production of infectious recombinant virus, while transfection into ETIF-expressing cell lines resulted in the generation of progeny virus (von Einem et al., 2006), indicating that ETIF is also necessary for the growth and replication of EHV; similar results were observed with BTIF (Misra et al., 1995).

**TABLE 1:** Compare the transcriptional activation ability of different HSV VP16 mutants and effects on virus proliferation.

HSV mutants	Mode of mutation	Changes in transcriptional activation of VP16	Effects on virus proliferation
SJO2	Ser375 substituted by Ala of VP16	Decreasing the transcription level of the IE genes.	A 10-fold reduction in viral titers.
In1814	Four amino acids inserts after residue 379 of VP16	Almost eliminating the transcriptional activation of VP16.	Plaque-forming efficiency of the virions is reduced by 100–10,000 fold.
RP5	Lacking of whole TAD of VP16	Completely inhibiting the transcriptional activation of VP16.	Plaque-forming efficiency and the viral titer decreases even more than in 1814.
8MA	Lacking of whole VP16	/	The virus cannot replicate in the non-complementary cells without VP16.
8MA-R	The revertant of 8MA	Completely restoring the transcriptional activation of VP16.	The virus fully reverts to normal levels of replication.

There are other new findings in the study of CHV and MDV. Infectious viruses cannot be produced after CHV genomic DNA is transfected into canine kidney cells unless the DNA is cotransfected with CHV VP16 (Tyack et al., 2006). MDV replicates in a highly cell-related manner in cell culture, and MDV can proliferate in specific feather follicle epithelium (FFE) cells of chickens but not in other cell cultures. Interestingly, VP16 is highly expressed in FFE cells (Dorange et al., 2002; Ponnuraj et al., 2019). These results show that VP16 is critical for the production of infectious virions by CHV and MDV. Moreover, exogenous VP16 is required to generate progeny viruses, which may be due to the inability of the cellular transcriptional activation system to activate viral genome transcription and its reliance on the transcriptional activation of IE genes mediated by the introduced VP16 to promote efficient genome expression in series. Contrary to the above studies, PRV VP16 is unnecessary for replication, and the VP16 null virus PRV-ΔVP16 can still proliferate in host cells. Although the virus grows slowly, the plaques are small, and the viral titer is significantly reduced, these defects can be fully recovered in PRV VP16-expressing cell lines (Fuchs et al., 2002, 2003). An ORF10 deletion mutant of VZV also proliferates normally in cell culture, and the viral titer is not decreased significantly, indicating that ORF10 is also unnecessary for VZV replication (Cohen and Seidel, 1994).

### Influencing the Formation of Mature Virions by VP16

#### Effective Integration of VP16 Into the Tegument Requires VP1/2

**FIGURE 2:**
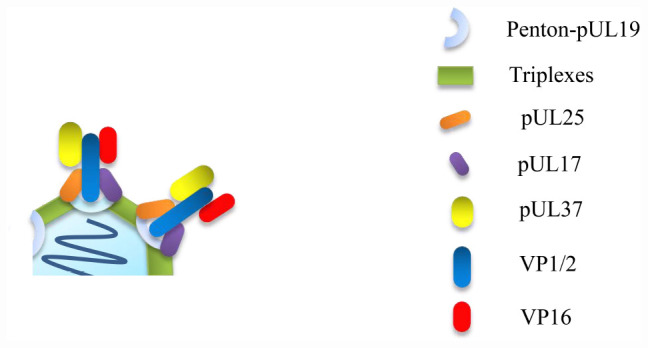
VP16 cannot be directly attached to the capsid alone, effective binding of VP16 to capsid requires VP1/2, and VP16 cannot only promote the formation of assemblies, so as to promote the maturation of nucleocapsids, but also promote the acquisition of outer tegument and envelope of nucleocapsids in the cytoplasm (Owen et al., 2015; Heming et al., 2017).

In which stages does VP16 mainly participate in the virion assembly process? What interaction networks are involved? Observing PRV particles, such as most tegument proteins, VP16 can be detected only in nucleocapsids in the cytoplasm or extracellular mature virions, and it is integrated into nucleocapsids before secondary envelopment in the cytoplasm (Fuchs et al., 2002, 2003). However, in HSV-1, VP16 can be detected in the perinuclear space, indicating that it is integrated into nucleocapsids before primary envelopment (Mossman et al., 2000). HSV-1 VP16 can interact with some capsid proteins, such as pUL17, pUL19, pUL25, and the complex of pUL18–pUL38, so that VP16 can bind to the assembly site of immature capsids to promote the formation of assemblies (a nuclear structure formed by aggregation of immature capsids and some tegument proteins), thus promoting the maturation of nucleocapsids (Ward et al., 1996; Dai and Zhou, 2018). However, VP16 cannot bind to capsid alone, and the effective assembly of VP16 on the capsid is dependent on the largest tegument protein VP1/2 (encoded by UL36) (Coller et al., 2007; Svobodova et al., 2012) (Figure 2).

**FIGURE 3:**
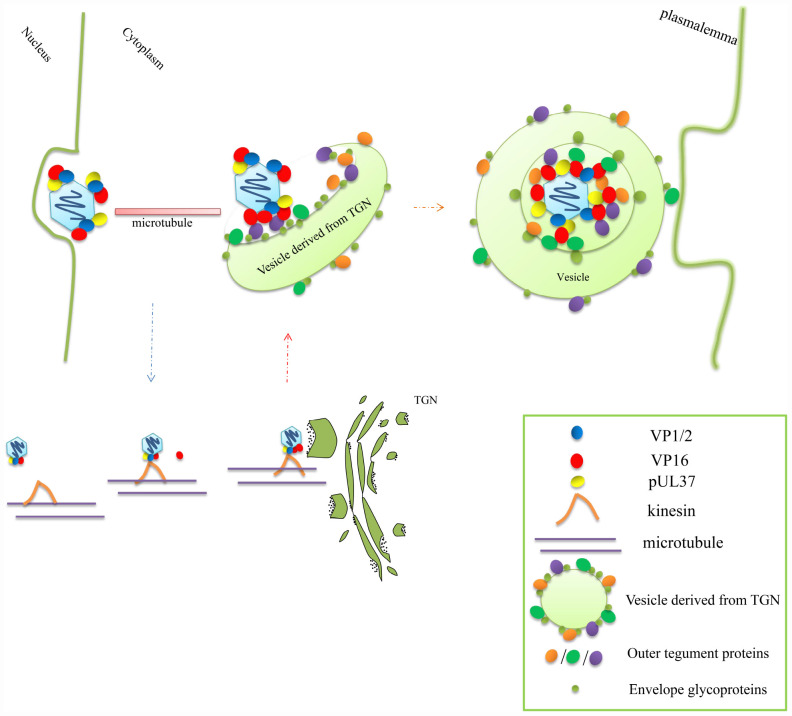
VP16 promotes nucleocapsid microtubule-transport and secondary envelopment in the cytoplasm. The complex composed of VP16, VP1/2, and pUL37 can bind kinesin on the microtubule to promote nucleocapsid transport; during transport, the capsid particle can bind to more VP16. When the nucleocapsid is transported to the specialized vesicles, which derived from the TGN, that are studded with various outer tegument proteins and glycoproteins, the nucleocapsid can effectively bud into the vesicle through the interaction between the tegument proteins and glycoproteins and thus obtain secondary envelopment. The secondary envelopment step also provides a transport vesicle that later fuses with the plasmalemma to release the mature enveloped virion out of the cell (Kelly et al., 2009; Owen et al., 2015).

VP1/2 can bind to nucleocapsids in the nucleus, facilitating the primary envelopment and nuclear egress of the capsids (Schipke et al., 2012; Ivanova et al., 2016). VP1/2 can directly interact with VP16 to guide the capsids to the binding site of VP16; therefore, VP16 is assembled on the nucleocapsids. HSV VP16 begins to bind to capsids via VP1/2 in the nucleus (Ko et al., 2010; Svobodova et al., 2012). After the nucleocapsids leave the nucleus, additional VP16 can integrate into the tegument so that the number of VP16 molecules in the extracellular mature HSV virions is much higher than that in the enveloped primary virions in the perinuclear space. However, PRV VP16 binds to capsids only in the cytoplasm via VP1/2 (Naldinho-Souto et al., 2006). VP1/2, VP16, and another inner tegument protein, pUL37, can form a complex under the mediation of VP1/2 to promote the transport of capsids along microtubules in the cytoplasm, although pUL37 does not directly interact with VP16 (Sandbaumhüter et al., 2013; Laine et al., 2015) (Figure 3). Moreover, in HSV-1, the levels of VP1/2 and pUL37 seem to be strictly controlled and do not change even in the absence of VP16; however, there is a compensation relationship between VP16 and pUL37 in PRV, as a decreased quantity of VP16 will lead to an increase in the introduction of pUL37, and a lack of VP16 can also be compensated by the addition of other tegument proteins, such as VP11/12 and VP22 (encoded by UL49), or cellular proteins, such as dynein, which may be the main reason why VP16 is not an essential protein for PRV replication (Naldinho-Souto et al., 2006; Bucks et al., 2011; Diefenbach, 2015).

### VP16 Affects the Maturation of Virions Mainly by Promoting the Secondary Envelopment of Nucleocapsids

Although PRV-ΔVP16 can still produce a low level of infectious virions, the morphology of the virions is severely deficient in that the newly formed nucleocapsids are largely retained in the cytoplasm, while few enveloped virions are found in the cytoplasm or extracellular space; in contrast, a large number of capsid-free particles are produced and released (Fuchs et al., 2002, 2003). The morphology of EHV virions with ETIF deletion also has similar defects: The nucleocapsids can start budding on Golgi-derived vesicles but cannot completely enter the vesicles. There is a blurry mass around the nucleocapsids, which may block the secondary envelopment of the nucleocapsids (von Einem et al., 2006). After the HSV VP16 deletion strain infects cells, similarly, a large number of non-enveloped capsids accumulate around the vesicles, which are unable to enter the vesicles and produce extracellular enveloped particles. Enveloped virions can be observed only in the perinuclear space, and the quantity increases significantly; moreover, the level of DNA encapsidation decreases and the number of empty capsids increases significantly (Mossman et al., 2000; Knez et al., 2003). This also confirms that there are some interactions between VP16 and capsid proteins in HSV that can promote DNA encapsidation (Table 2). It also shows that VP16 has an impact on the egress of primary enveloped particles from the perinuclear space, but the effect is not significant. The most important factor causing the failure of virions to mature due to the absence of VP16 is that the secondary envelopment is severely blocked, and the nucleocapsids cannot enter the vesicles; as a result, they cannot be excreted from the cell through vesicle transport and fusion with cell membranes (Yuan et al., 2005; Diefenbach, 2015; Owen et al., 2015).

**TABLE 2:** Comparison of virus replication and morphology of different VP16 deletion strains.

Different VP16-null mutants	Proliferation and replication of these VP16 deleted mutants	The morphology of these VP16-deleted mutants
HSV-ΔVP16	The virus cannot produce offspring, and its growth is suppressed in the non-complementary cells without VP16.	Unenveloped capsids accumulate around the vesicles, and the enveloped virions can only be observed in the perinuclear space; the level of DNA capsidization decreases, and the number of empty capsids increases significantly.
PRV-ΔVP16	The virus grows slowly, the plaque gets smaller, and the viral titer is significantly reduced.	Few enveloped virions in the cytoplasm or extracellular space; in contrast, a large number of capsid-free particles are produced and released, and unenveloped nucleocapsids are largely retained in the cytoplasm.
EHV-ΔETIF	The virus cannot replicate in the non-complementary cells without ETIF.	The budding of nucleocapsids on Golgi-derived vesicles is incomplete and cannot completely enter the vesicle, and there is a fuzzy mass around the nucleocapsids.
BHV-ΔBTIF	The virus cannot multiply effectively in the non-complementary cells without BTIF.	Similar to the morphology of EHV-ΔETIF, unenveloped nucleocapsids are largely retained in the cytoplasm, and the secondary envelope was suppressed.
VZV-ΔVP16	The virus also proliferates normally in cell culture, and the viral titer is not decreased significantly.	The morphology of the virus is normal; multiple enveloped intact virions also be released to the outside of the cell.
		

### The Mechanism of VP16 Effects on Secondary Envelopment

**FIGURE 4:**
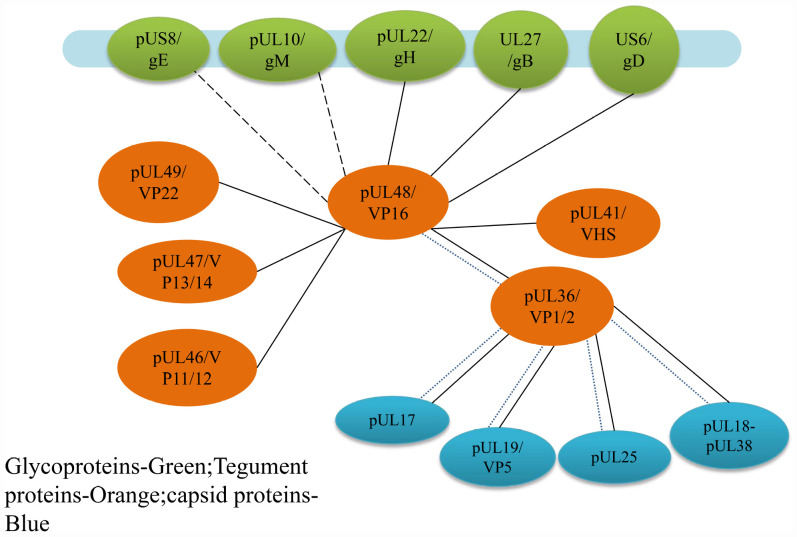
Protein interaction networks of VP16 that are involved in the assembly of alphaherpesvirus. It is the important linker from capsid-associated proteins to envelope-associated proteins. Solid lines indicate direct interactions, and short dashed lines show indirect interactions with capsid proteins by VP1/2 demonstrated in HSV, and the long dashed lines demonstrated in PRV (Owen et al., 2015).

How does VP16 affect the secondary envelopment of nucleocapsids? Through the analysis of the particle composition of nucleocapsids in the cytoplasm and extracellular capsid-free particles of VP16-null HSV and PRV, the tegument components of these two types of particles were found to be different. The tegument proteins on the nucleocapsids in the cytoplasm are mainly the inner proteins VP1/2 and pUL37, while the tegument components of extracellular capsid-free particles do not contain VP1/2 and pUL37 but mainly the outer tegument proteins VP11/12, VP13/14, and VP22 (Fuchs et al., 2002, 2007; Laine et al., 2015). This finding indicates that the connection between the inner tegument proteins and the outer tegument proteins is severed in the absence of VP16 and that VP16 acts as a bridge between the inner tegument and outer tegument. The large number of capsid-free particles indicates that the involvement of the outer tegument and Golgi-derived envelope in the maturation of virions is not affected by the nucleocapsid and proves that the formation of a whole virus particle is actually composed of two parts: the nucleocapsid and the outer tegument–envelope complex present on the vesicles derived from the *trans*-Golgi network (TGN) (Mettenleiter et al., 2006; Owen et al., 2015; Crump, 2018). VP16 is the key to the effective combination of these two parts (Figure 3). VP16 binds to the nucleocapsid through the action of VP1/2. When nucleocapsid is transported to the secondary envelope loci that associate with the Golgi apparatus, VP16 cannot only bind to outer tegument proteins of the outer tegument–envelope complex but also directly bind to some glycoproteins of the complex (Figure 4). After contact with the glycoproteins, the produced nucleocapsids can effectively bud in vesicles to complete secondary envelopment; this is because the glycoproteins can identify specific receptors on the membrane and promote the penetration of virions (Arii and Kawaguchi, 2018; White et al., 2020).

### Interactions of VP16 With Inner Tegument Proteins

**FIGURE 5:**
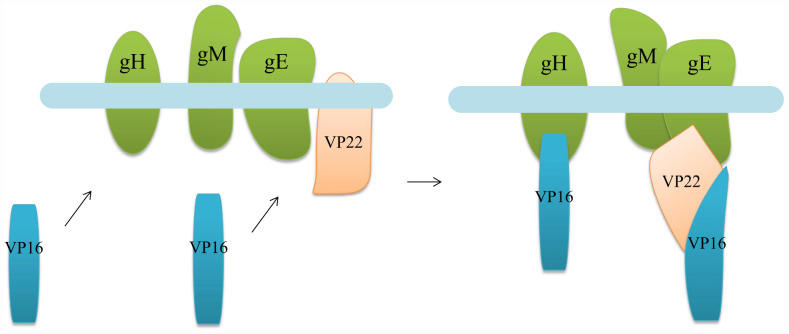
Diagram illustrating the binding of VP16 to gH and binding to the envelope through VP22. When the nucleocapsid is transported to the vesicle derived from the Golgi membrane, VP16 on the capsid cannot only directly bind to some glycoproteins on the envelope but also combine with the envelope through some tegument proteins to promote the secondary envelope of the nucleocapsid (Diefenbach, 2015; Owen et al., 2015).

The main outer tegument protein that interacts with VP16 at this stage is VP22. In HSV-1, VP22 effectively assembles into the nucleocapsid, which requires a collective effect by the VP16-binding region at residues 160–212 and the cytoplasmic complex-binding region at residues 213–301 at the C-terminus. Without the VP16-binding region, the amount of VP22 integrated into the tegument is significantly reduced, but VP22 does not inversely affect VP16 recruitment (Hafezi et al., 2005; O’Regan et al., 2007; Hew et al., 2015) (Figure 5). In addition to improving the efficiency of IE gene transcription mediated by VP16, the other two tegument proteins VP11/12 and VP13/14 can also promote secondary envelopment (Murphy et al., 2008; Guo et al., 2010; Owen et al., 2015). However, in this process, the relationship between VP11/12, VP13/14, and VP16 needs to be further explored.

#### Interaction of VP16 With Envelope Glycoproteins

The interaction between VP16 and envelope glycoproteins is not always the same in different alphaherpesviruses. In HSV-1, VP16 can bind to gH, and the reaction between the two belongs to the association between the tegument polypeptide and cytoplasmic tail of the envelope glycoprotein (Figure 5), providing the molecular driving force for nucleocapsid budding at the vesicle membrane (Gross et al., 2003). This association is temperature dependent, taking place at a physiological temperature of 37°C but not at low temperatures. NMR analysis shows that this effect is related to the conformation of the C-terminal peptide of gH. In a low-temperature environment, the gH peptide forms a specific and stable structure, while at physiological temperature, the peptide structure disintegrates and becomes irregular in order to bind to VP16. This effect raises the hypothesis of the “induced fit” mechanism of gH, that its tail is plastic and unstructured before binding, so that it can interact with VP16 and form a specific conformation, while another conformation may form when it binds to other tegument proteins (Gross et al., 2003; Kamen et al., 2005). In addition, in HSV-1, VP16 can also interact with gB and gD, but whether the interaction modes are the same as that of gH has not been studied (Johnson et al., 2011; You et al., 2018; Komala Sari et al., 2020). In PRV, VP16 interacts mainly with gM and gE but not with the cytoplasmic tail of gH (Chang et al., 2009; Omar et al., 2013). Although the HSV-1 and PRV gH proteins are homologous, the cytoplasmic tail of gH is extremely unconserved. It is possible that the cytoplasmic tail of PRV gH does not have a structural basis for binding to VP16 but can interact with other tegument proteins and participate in secondary envelopment.

### The Negative Regulation of VHS by VP16 Also Affects Viral Assembly

In addition to facilitating transcription of the viral genome and secondary envelopment of nucleocapsids, VP16 can also affect viral assembly through the negative regulation of host shutdown protein VHS (encoded by UL41) (Knez et al., 2003). VHS can non-selectively degrade host cellular and viral mRNA, and when the host cellular mRNA is degraded, VP16 can combine with VHS to inhibit its mRNA degradation activity and accumulate viral mRNA, which can stimulate viral gene expression (Knez et al., 2003; Strand and Leib, 2004). After HSV VP16-null strain 8MA infection of cells, VHS activity is unrestricted due to the inability to synthesize VP16 and results in excessive degradation of viral mRNA so that its translation is aborted halfway through the infection cycle, leading to the inhibition of synthesis of a variety of viral proteins. The failure of the normal synthesis of multiple structural proteins inevitably leads to a serious impact on viral assembly. For example, the expression of the capsid protein pUL19 is inhibited, which also leads to a reduction in the level of DNA encapsidation and an increase in the number of intracellular and cytoplasmic empty capsids (Heming et al., 2017). Therefore, it is reasonable to suspect that the unrestricted activity of VHS is the main reason why 8MA cannot replicate effectively. The study of the VP16 and VHS double-deleted strain 8MA/ΔSma answers this question. Compared with 8MA, 8MA/ΔSma shows no difference except increased encapsidation of DNA, and the virus can still not proliferate in cell lines without VP16 expression (Mossman et al., 2000). This finding indicates that the fatal defect of virus replication caused by VP16 deficiency cannot be overcome by inhibiting the expression of VHS; it is mainly because the connection between the nucleocapsid, outer tegument, and envelope is cut off, and effective secondary envelopment cannot be carried out. There is no significant difference in the morphology of nucleocapsids and mature virions of the ORF10-null VZV strain precisely because, in VZV, ORF10 is unnecessary to guide the secondary envelopment of the virions, so ORF10 is not important for VZV maturation, which may be dominated by other tegument proteins (Cohen and Seidel, 1994; Cai et al., 2012). Although the life cycles of different alphaherpesviruses all go through similar processes, the viral proteins involved or playing major roles at different stages and the specific molecular mechanisms may vary, and additional research is needed to elucidate the specific processes of the life cycles of different alphaherpesviruses.

## *De Novo* Synthesis of VP16 Coordinates Reactivation From HSV Latency

Latent infection is a characteristic of herpesviruses and can help them evade the host immune response. Once the virus establishes latent infection, its genome can persist in the cell and may be activated by external factors to reenter the lytic period of replication and proliferation. Neurocytes are the primary site of latent herpesvirus infection (Kollias et al., 2015; Menendez and Carr, 2017). HSV establishes latent infection only in the postmitotic neurons of the host peripheral nervous system. Under the periodic stimulation of different stressors, the latent state of infected neurons is activated and then enters the lytic cycle to regenerate infectious virions (Kennedy et al., 2015; Whitley and Baines, 2018; Rübben, 2020). The establishment of latent infection is related to the interaction between the viral genome and the nuclear environment (Zhang et al., 2020). The viral genome and promyelocytic leukemia (PML) protein occupy the same nuclear region in infected neurons and eventually form nucleosome structures containing viral DNA (PML-NBs) (Cohen et al., 2018; Watson et al., 2018). ICP0 can induce degradation of a variety of cellular proteins through the proteasome pathway, including components of PML-NBs and centromeres. Thus, ICP0 can induce the destabilization of PML-NBs and centromeric chromatin, contributing to the establishment of a suitable nuclear environment for lytic infection (Lomonte and Morency, 2007; Gross et al., 2012; Wang et al., 2012), and ICP4 plays a major regulatory role in gene transcription and is necessary for HSV to undergo lytic infection (Hu et al., 2019). Thus, ICP0 and ICP4 are the two main proteins involved in the activation of HSV latent infection. Earlier studies suggested that VP16 has no effect on the exit of alphaherpesvirus from latency and the initiation of the lytic cycle because it is difficult to rely on structural proteins produced at the late stage of the infection cycle to initiate transcription of the viral genome during the lytic phase (Sears et al., 1991; Ecob-Prince et al., 1993). However, an increasing number of studies indicate that VP16 plays a significant role in HSV reactivation from latency (Thompson et al., 2009; Danaher et al., 2013; Sawtell and Thompson, 2016). VP16 may not be efficiently transported to the cell body through the axon, resulting in suppressed synthesis of ICP0 and ICP4, which promotes the entry of virus into latency (Thompson and Sawtell, 2010; Sawtell et al., 2011). Once VP16 is deficient, latently infected TG neurons may not detect the expression of viral proteins after stress stimulation. However, in the absence of other viral proteins, the VP16 promoter can be activated in neurons that are latently infected after stress stimulation (Thompson et al., 2009). When the virus changes from latency to the lytic cycle, VP16 does not act the same as the typical model of a late gene; it has the ability to be synthesized before IE genes in the initial stage of reactivation from latency and does not depend on ICP0 and viral DNA replication. This phenomenon is dependent on the Egr-1/Sp1 sites on the VP16 promoter near the VP16 TATA box, which is responsible for regulating *de novo* synthesis of VP16 in the initial stage of reactivation from latency. Viral DNA replication in infected neurons is initiated by *de novo* VP16 synthesis induced by the Egr-1/Sp1 sites (Diefenbach et al., 2008; Thompson et al., 2009; Thompson and Sawtell, 2010, 2019). Moreover, when VP16 can be effectively transported to the cell body of neurons, the expression of the IE genes ICP0 and ICP4 can be regulated by the TAATGARAT element by VP16, thereby causing lytic infection (Thompson and Sawtell, 2019). If the transcriptional activation function of VP16 is affected, such as when Ser375 is replaced by alanine, the number of neurons that can reexpress viral proteins in latently infected neurons during heat shock treatment is significantly reduced (Sawtell et al., 2011), indicating that the transcriptional activation function of VP16 is crucial for HSV reactivation from latency. However, VP16 is not unique to alphaherpesvirus reactivation from latency.

After stress stimulation, the latent infection established by BHV-1 can be reactivated even in the absence of VP16 (Kook et al., 2015). This phenomenon can occur because stress stimuli can increase the level of corticosteroids, such as glucocorticoid (GR), and there are glucocorticoid receptor response sites (GREs) upstream of the IE gene enhancer that can bind to GR. These sites are not related to the core motifs that are specifically recognized by VP16; moreover, in the absence of VP16, HCF-1 can combine with GR to form a complex that then acts on GREs to promote the transcription and expression of IE genes, thus promoting lytic infection (Frizzo da Silva et al., 2013; Kook et al., 2015; Sawant et al., 2018). Although no related reports were found in HSV, this observation proposed another mechanism for the reactivation of herpesviruses from latency, suggesting that VP16 is important for reactivation from latency but not unique. There may be other unknown mechanisms to promote herpesvirus reactivation from latency that require further investigation, especially cell factors such as HCF-1, which are particularly critical, requiring special attention.

## Conclusion

In alphaherpesviruses, VP16 is a powerful transcriptional activator that specifically acts on IE genes, and the transcriptional activation mechanism of HSV VP16 has been studied most deeply. In addition to the formation of transcriptional regulatory complexes with HCF-1 and Oct-1, it is not clear whether VP16 has other mechanisms of regulating IE gene transcription. The current research focuses on the interaction of VP16 with other cellular or viral proteins and the mechanism and structure of the TAD associated with target proteins, which can help us understand how the TADs of other transcriptional activators work. Moreover, the transcriptional activation mediated by VP16 also plays an important role in the reactivation of alphaherpesviruses from latency. VP16 is also one of the main tegument components of alphaherpesviruses, and it is the interface between capsid-associated proteins (such as VP1/2) and membrane-associated proteins (such as VP22). In the secondary envelopment of nucleocapsids, VP16 plays the important role of “bridging,” effectively promoting capsid envelopment and mature virion formation. Although there have been many studies on the involvement of VP16 in the envelopment and assembly of virions, the interaction between VP16 and proteins involved in this process needs further exploration. Additionally, what different reactions will happen in alphaherpesviruses in which VP16 is not critical for assembly needs to be studied further, which will not only give us a clearer knowledge of VP16 but also be important for a deeper understanding of the alphaherpesvirus life cycle.

## Author Contributions

All authors listed contributed to the completion of the article. DF and MW contributed to the design and wrote the article. RJ, QY, YW, DZ, XZ, SC, ML, SZ, XO, SM, QG, and DS provided ideas contributing to the conception of this article. XW, YL, YY, LZ, LP, and XC helped to create the tables and figures. AC modified the article. All authors reviewed and approved the final manuscript.

## Conflict of Interest

The authors declare that the research was conducted in the absence of any commercial or financial relationships that could be construed as a potential conflict of interest.
